# Designing a video consultation area for hybrid care delivery: the Garden Room with a view

**DOI:** 10.3389/fdgth.2023.1198565

**Published:** 2023-07-25

**Authors:** Merlijn Smits, Demi van Dalen, Danny Popping, René Bleeker, Martijn W. J. Stommel, Harry van Goor

**Affiliations:** ^1^ PBLQ, Den Haag, Netherlands; ^2^ Department of Surgery, Radboud University Medical Center, Nijmegen, Netherlands; ^3^ Radboud University Medical Center, Nijmegen, Netherlands

**Keywords:** video consultation, evidence-based healthcare design, hybrid care, built environment, human factors

## Abstract

**Introduction:**

Accelerated by the coronavirus pandemic, the healthcare landscape is rapidly evolving, with a shift towards hybrid care models combining in-person and online care. To support this shift, the Radboudumc, an academic hospital in the Netherlands, decided to redesign an existing space facilitating the conduction of video consultations.

**Method:**

The design process involved participation of end-users to ensure that the physical space met their needs. The look and feel of the area was based on evidence-based design guidelines. Two prototype setups were built and tested, and the feedback informed the final design of the Garden Room.

**Results:**

Identified end-user needs were divided into 3 major categories entailing consultation room setup, optimal use of technology and practical issues involving room availability. Combined with the look and feel of the hospital, final design requirements were developed. The Garden Room consists of 18 video consultation rooms, 4 shared workspaces, relaxation area with kitchen, and meeting rooms. Specific attention is given to the ergonomics, technology and privacy in the rooms to facilitate optimal video conversations between patients and healthcare providers. In the Garden Room, natural elements and an open design supports working in a healing environment.

**Discussion:**

Next challenge will be optimizing the use of the Garden Room, which may be hindered by various barriers like resistance to change, existing work processes, and lack of skills training. To address these barriers and support use of the Garden Room, the hospital should focus on the implementation of education, changes in work processes, and the presence of advocates for telehealth.

## 1. Introduction

The healthcare landscape is rapidly evolving, with a shift towards hybrid care models that combine in-person and online care (1). One aspect of this shift is the increasing use of video consultations as alternative to traditional in-person visits. Video consultation enables the healthcare professional to provide care remotely from virtually any location through real-time interaction with patients through a videoconferencing system using a webcam, smartphone or tablet on both sides (2). Video consultations have shown similar results to in-person communication with the benefits of reduced travel time and costs, lower carbon emission, shorter wait times, and greater accessibility and participation for patients (1, 3–6). With these promising effects and pandemic-related adoption, video consultation is considered as a major method of future care provision (7).

With these developments, also hospitals are increasingly applying video consultations in their current environment. Incorporating in-person and online care into a single healthcare facility requires rethinking and redesigning the physical environment, meeting the needs arising from the application of video consultations of both patients and healthcare professionals. This may, for example, include the implementation of quiet rooms for video consultations within the existing facility to minimize hindering back ground noises during the conversation between the patient and healthcare professional. In addition, designing a video consulting experience requires attention for the virtual experience. The online background should, for example, be free from distractions and provide facility identification for an optimal focus on the conversation.

In the design of the built and online environment, it is important to consider “human factors” by tailoring the environment to the needs, both physical and cognitive, of the end-users (8). For that, the movement of evidence-based design (EBD) has emerged as “a process for creating healthcare buildings, informed by the best available evidence, with the goal of improving outcomes” (9). Although EBD generally focuses on the physical environment, its’ theory is also applicable to the design of an online and hybrid space (10, 11). To apply evidence-based design, one needs to review existing literature and/or conduct empirical research to obtain knowledge about the creation of healthcare environments that are optimally aligning to user needs and generating best healthcare outcomes. Design decisions are consequently made based on the best available knowledge (12). Empirical research methods for studying user needs in relation to healthcare design include participatory design by involving end-users in the process and conducting usability testing before implementation (8, 13, 14).

In this article, we describe the evidence-based design process of a video consultation area at the Radboudumc, an academic hospital in The Netherlands, Nijmegen. The Radboudumc is increasingly supporting hybrid care, including video consultations. The hospital foresees that healthcare delivery in the future will be largely remote. To support this shift, the built environment of the hospital is based on the vision of “less bricks, more bytes, different behavior” (15). Designing a video consultation area matches this vision. An existing atrium, originally used for meetings and conferences, known as the “Garden Room” (Dutch: *Tuinzaal*), was designated as a central area for video consultations. Here, we discuss the process of redesigning this area, considering needs of both patients and healthcare professionals such as control of environmental distractions and adjustment of audio and video facilities, and the look and feel of the Radboudumc as an input for the evidence-based design process. We conclude with a discussion on how to optimize the use of the video consultation area in the future.

## 2. Methods

The design processes of the Garden Room were supported by the Hospitality Group, a design consultancy group in Amersfoort. To ensure that the design of the video consultation area was tailored to the needs of the end-users—healthcare professionals providing remote care—interviews were conducted, design requirements developed, and mock-up prototypes tested. Healthcare professionals for the interviews were selected by purposive sampling. Four medical professionals who frequently use video consultations and one member of support staff involved in the implementation of video consultations were asked to reflect on their work and needs. These professionals included a medical oncologist, a surgeon, a plastic surgeon, a pediatric pulmonologist and administrative planner (two men, three female). The interviews were conducted at the beginning of the design process by members of the design consultancy group. Questions revolved around the setup of equipment in the pod, the interior of the pod, technical requirements and requirements for conducting multidisciplinary consultations, optimization of the use of the pods ad a reservation system, important ergonomic conditions and any other essential facilities that should be considered in the Garden Room.

All interviews lasted approximately 1 h and provided insights into the needs and preferences of the end-users. The collected information was presented to 11 end-users with varying specialties during two brainstorm sessions. Expressed needs were explained and additional questions were asked until a complete overview of the end-user's needs had been established. In addition to these user needs, also the need to align the design of the video consultation area with the look and feel of the Radboudumc was considered. Together, this resulted in a list of design requirements for the video consultation area.

Based on this list of design requirements, we built and tested two prototype setups. Five end-users tested the prototypes using an *ad hoc* developed evaluation form addressing (1) IT; (2) Acoustics; (3) Indoor climate; (4) Light; (5) Ergonomics; (6) Ambience; (7) Background view. The feedback from these evaluations informed the final design of the Garden Room.

## 3. Results

**Figure 1:**
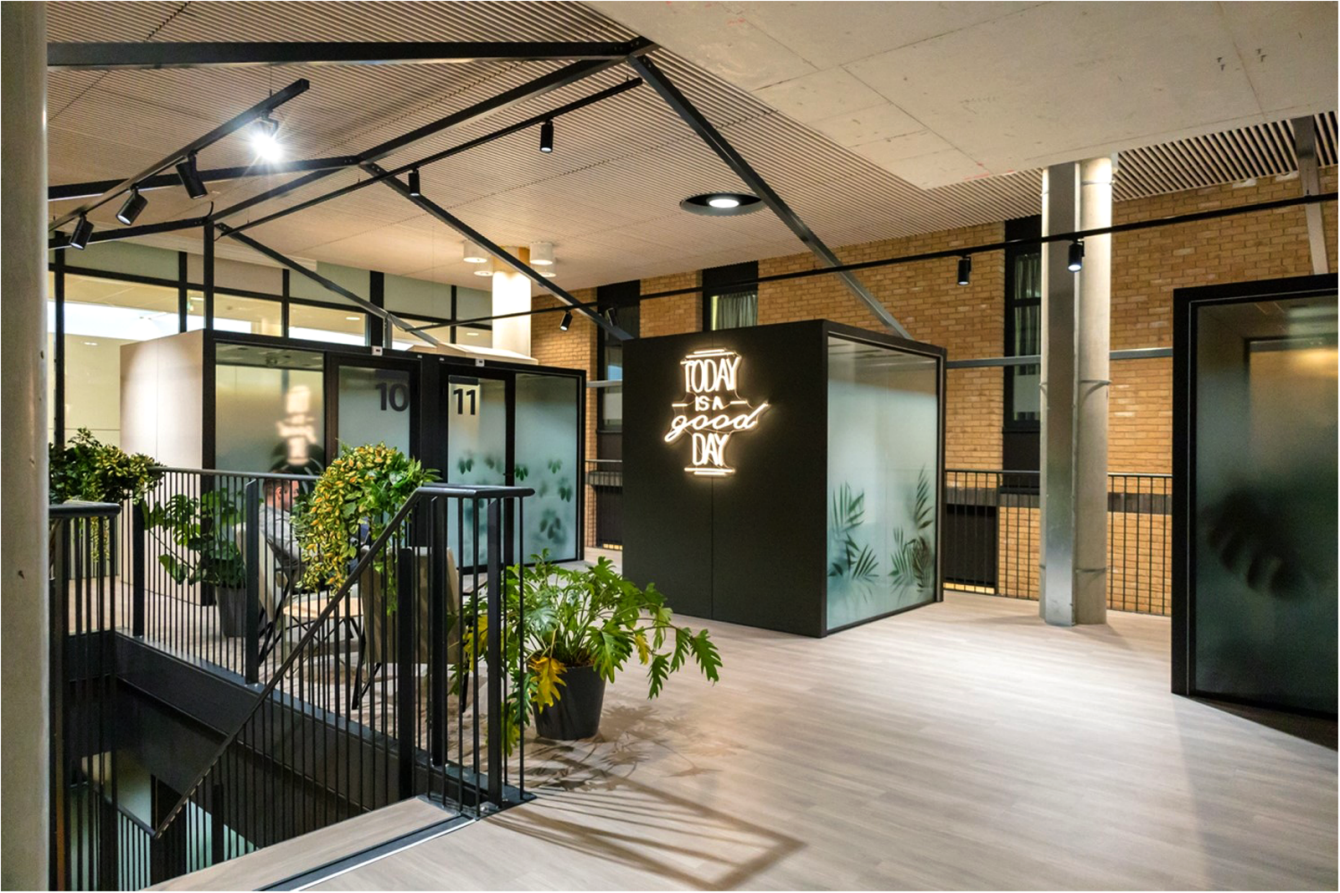
Impression of the second floor of garden room. Photographer: Rylana van der Marel, Hospitality Group.

The end-user needs retrieved from the interviews and brainstorm sessions, combined with the look and feel of the hospital, eventually resulted in a list of design requirements which were the guiding principle for the design of the Garden Room. The video consultation area was designed and built between December 2020 and June 2022. Figure 1 provides an impression of the second floor of the area with a few consultation pods.

### 3.1. End-user needs

The identified needs from the end-users were categorized in three domains; consultation room set-up, technology, and room availability. The first category entailed requirements for a comfortable working space and efficient set-up to optimize the conversation. The second involved communication and electrical technology, the latter mainly practical conditions for scheduling a video consultation. An overview of the needs is given in Supplementary Table S1 and processed into a first sketch design, Supplementary Figure S2.

### 3.2. Spatial requirements

The Garden Room consists of a large space that has been divided into several sections: video consultation rooms, shared workspaces, a kitchen and relaxation area, and meeting rooms. A total of 18 video consultation rooms were installed using modular boxes, each of which can accommodate one or two healthcare providers -this latter is meant to supervise students. Each box is designed to be acoustically sealed for privacy of healthcare providers and patients during the consultation. Each box is further equipped with a desk with adjustable height, an ergonomic office chair, a dual screen display for showing both the video consult and the patient's electronic health record, a camera that can be adjusted to eye level of speaker, and sufficient charging outlets. These amenities are intended to facilitate efficient remote care consultations. The Garden Room is located in the center of the academic hospital. Healthcare providers can reserve the consultation rooms during the day via a reservation system, and presence sensors above the rooms indicate which rooms are occupied.

The four shared workspaces offer healthcare providers a place to do administrative work and are located near the video consultation rooms to prevent unnecessary occupation of these rooms for non-video activities. The kitchen and relaxation area offer healthcare providers the opportunity to take breaks between video meetings and, when desired, accessing the hospital garden. The Garden Room also includes several meeting rooms for private meetings between healthcare providers, such as preparing for a video consultation.

### 3.3. Look and feel of the surrounding hospital

**Figure 2:**
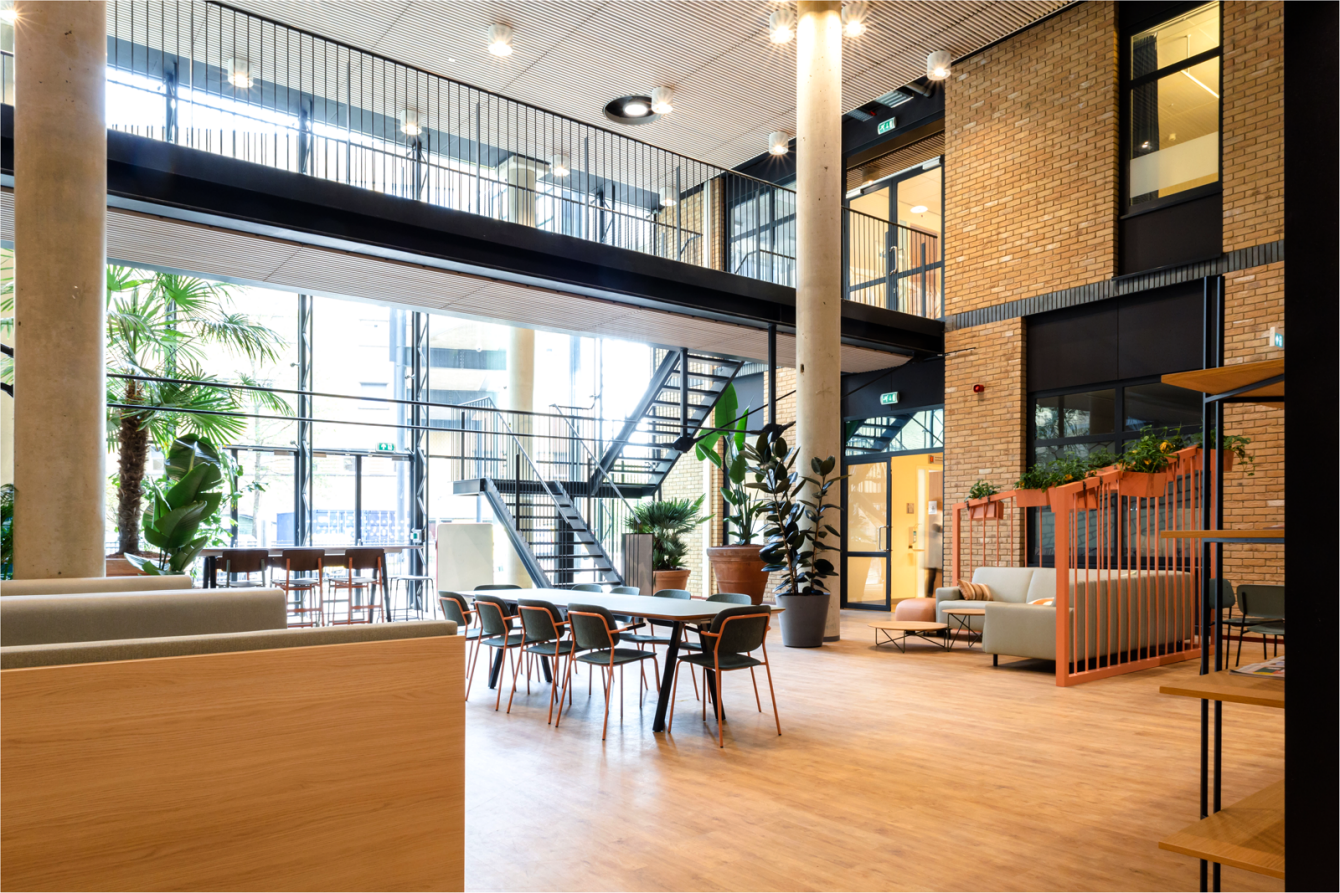
Impression of the relaxation area in the garden room with view on an outside terrace and garden. Photographer: Rylana van der Marel, Hospitality Group.

The design of the Garden Room follows the look and feel of the Radboudumc, adhering to six design guidelines: connection to nature, healthy living, innovation, openness and transparency, personalization, and professionalism. To foster a connection to nature, the Garden Room is not only providing direct access to a garden, but also incorporates many green and natural elements through the area, such as wood and greenery, combined with the use of a natural color scheme. Furniture is in most parts manufactured from recycled or recyclable material. The natural environment is intended to support relaxation and reduce stress, providing a healthy and healing environment for both patients and healthcare professionals. Additionally, to promote a healthy workspace, special attention has been given to the installation of a comfortable relaxation space and kitchen within the Garden Room, shown in Figure 2. This concept of hybrid working is in itself innovative and the Garden Room aims to express this innovativeness. The area is large and open, but also provides comfort and privacy through the use of modular enclosed boxes. Finally, by facilitating the right technologies for video consulting (dual screen, adjustable webcam), the hospital aims to support a personal and professional video consulting session between patients and healthcare providers.

**Figure 3:**
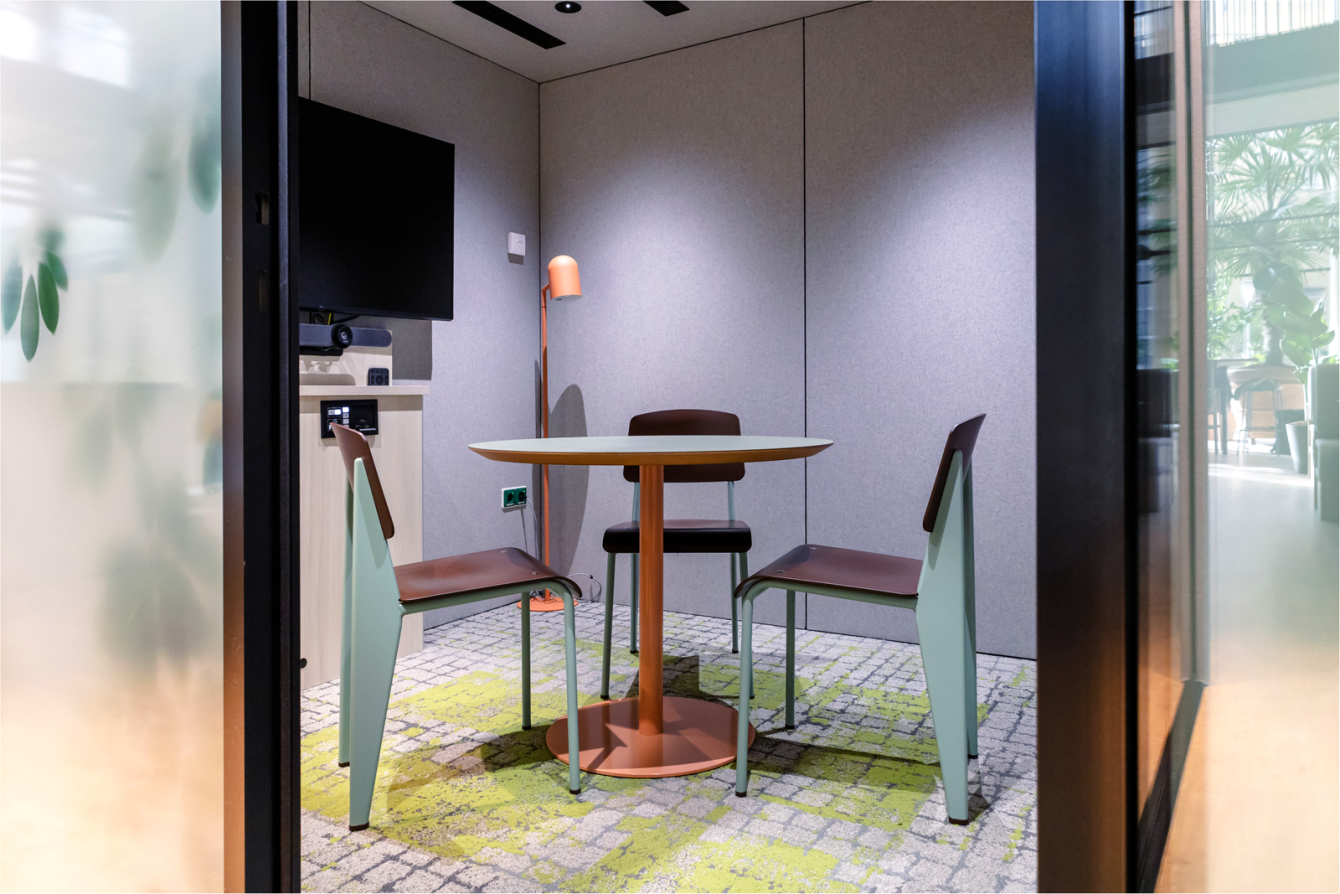
Impression of a video consultation pod for a multidisciplinary consultation. Photographer: Rylana van der Marel, Hospitality Group.

**Figure 4:**
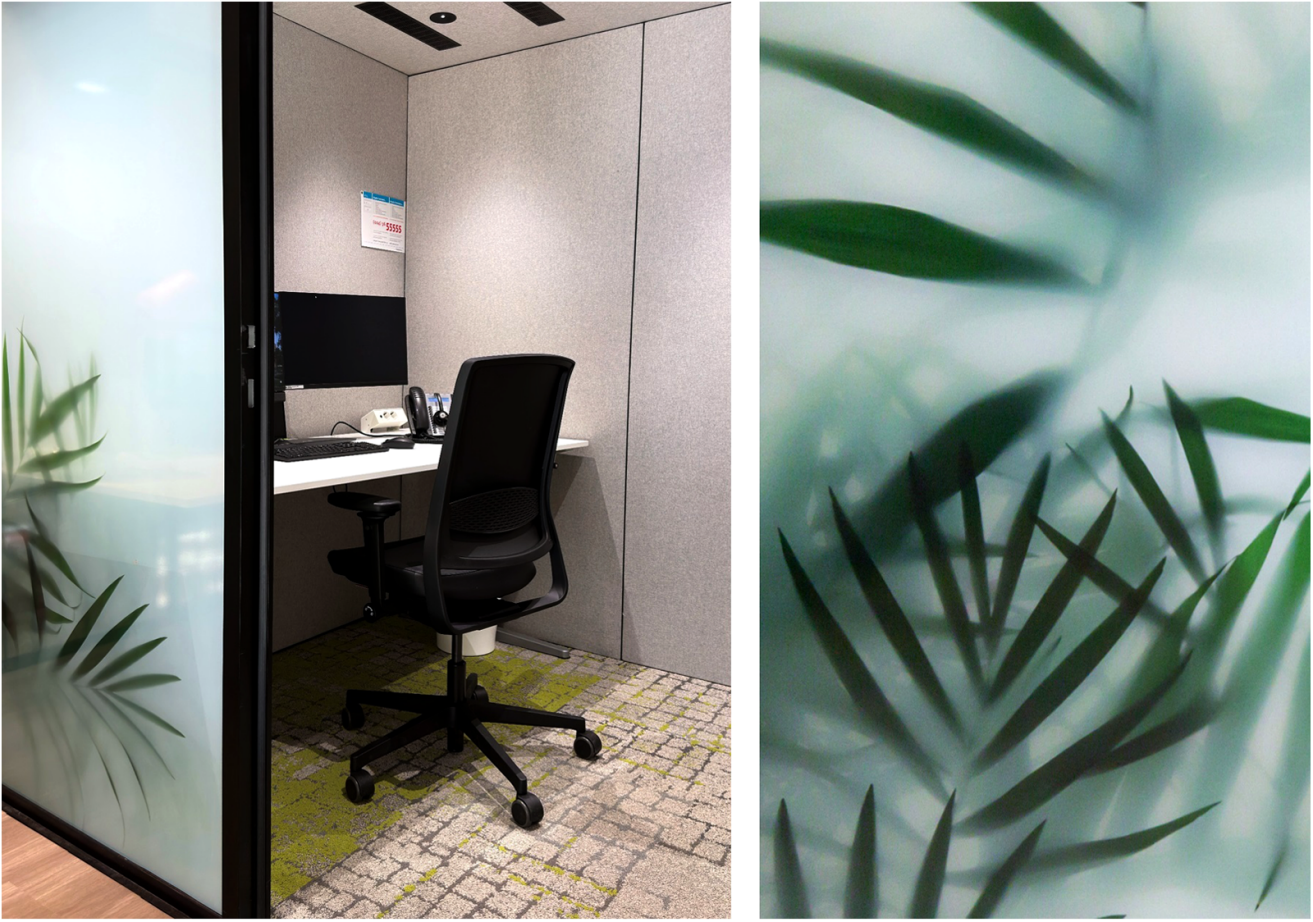
Impression of a video consultation pod. Photographers: collage of own material, Radboudumc (left), combined with greenery behind glass (right), Rylana van der Marel, Hospitality Group.

Within the modular video consultation boxes, the look and feel is well considered; patients virtually see their healthcare provider in the center of their screen, surrounded by the hospital's logo, warm and natural colors and plants. Adequate lighting creates a professional appearance. Out of the patient's view, a small window gives the healthcare provider an open and transparent appearance and allows others to quickly check if the box is occupied or not. A script, outside of the patient's view, is also provided to support the healthcare provider during the video consult. Figures 3, 4 give an impression of the interior of several pods.

## 4. Discussion

By considering the needs of end-users and the look and feel of the academic hospital, the Garden Room has become an open and natural space supporting hybrid care delivery. The room was put into service on July 1st 2022. Here we discuss how the design of the room is evidence-based and how use of the room can be scaled-up towards the future.

### 4.1. Design

Where possible, the Garden Room is based on best available scientific evidence for aligning physical and online space to the needs of end-users. The 2022 Guidelines for Design and Construction of Hospitals and Outpatient Facilities, for example, addresses design requirements for spaces used for telemedicine (16). One important aspect of video consultation is clear visibility during the consultation. Good visibility can enhance the relationship between the patient and healthcare provider and facilitate the demonstration of medical products or labels of medication. To improve visibility, it is recommended to use warm, white light (3,200–4,000 K), which was applied in the Garden Room (17). The color of the walls -and thus the background color in the video consult- should also be considered. A light blue color is generally recommended for its calming effect, which is consistent with the color of the hospital's logo provided in the Garden Room. To maintain focus, it is important to avoid clutter and distractions (18). The conditions for an optimal video consultation do not only apply to the area of the healthcare provider but also to the space of the patient. For an optimal conversation, patients can be informed about the location of their video call (17–19).

Furthermore, previous EBD research has shown that natural elements in the environment, such as plants and a view on trees, improve healthcare outcomes of patients and the overall mental well-being and cognitive functioning of the healthcare professional (19–21). For that, natural elements (e.g., plants, natural images) were used in the Garden Room to create such a “healing environment” (22). In addition, as exposure to daylight leads to better well-being of office workers due to its suggested antidepressant effect; each video box has a window to provide sufficient daylight for the healthcare professional (21, 23).

In addition to visual comfort, the right technology and audio quality are crucial to facilitate effective and trustworthy communication. In case of technical failure, it is recommended to have alternative forms of communication available (such as a phone) (4).

Finally, privacy is a major concern in video consultations. The Garden Room takes measures to ensure privacy through the use of acoustic sealing, opaque windows and the placement of normal windows only out of the patient's view (24).

### 4.2. Scaling-up

The Garden Room has recently been opened for use. In order to optimize the use of the Garden Room, there are several challenges that need to be addressed. The Garden Room is located centrally in the hospital in close in close proximity to the daily workplace of the healthcare professional as pointed out as relevant by the end-users (19). However, post-occupancy evaluations are necessary to determine whether the location of the Garden Room is indeed optimal for healthcare providers.

Another barrier to the adoption of video consulting is resistance to change (1). Many healthcare professionals are hesitant to adopt new technologies, as they may be unfamiliar with them or may prefer the personal interaction of in-person consultations (25). In order to overcome this barrier, it will be necessary to provide training and education on the use of video consulting technologies, as well as support for the integration of these technologies into the work processes of healthcare professionals. Additionally, given the high turnover of healthcare professionals there is a constant influx of new staff who are not familiar with the technology. Therefore, it is recommended to periodically consider education and training (4).

So far, research mostly focused on the design of the physical space. Further research should consider the behaviour and work processes within the Garden Room to optimize adoption of video consulting. This could be achieved through post-occupancy evaluation by, for example, combining a satisfaction survey with interviews and quantitative field data collection. The survey and interview should comprise questions about how the formerly end-users’ needs are met and about their experience with other technical and functional aspects of the consultation room area, such as privacy and comfort (26). Subsequently, key variables as room occupation and frequency of technical issues could be measured to provide a quantitative understanding of performance of the area as experienced by the healthcare professionals. Relevant would be to measure the (virtual) patient experience with the video consultation in this current setting as well.

Finally, to further facilitate the adoption of video consulting, there may be a need to encourage and give audience to advocates for telehealth within the organization (7).

### 4.3. Strengths and limitations

The Garden Room is a unique area for video consultations within the built hospital environment. It shows that an existing space can successfully be transformed into a hybrid care environment. Part of the successful design of the Garden Room is that end-users were involved in the design process and human factors were closely taken into consideration. However, only few end-users were interviewed, as only those who already advocated the use of telehealth were included. It would have been valuable to also include the perspectives of those healthcare professionals who may resist the implementation of telehealth to increase the adoption towards the future.

When successfully combining the post-occupancy evaluation results with the incorporation of behavioural and organizational improvements, the Garden Room will not only facilitate its function towards video consultation but also has the potential to be expanded in the future to include other forms of telehealth, such as remote monitoring of hospital and home-based patients.

## 5. Conclusion

The Garden Room exemplifies how the built environment can facilitate new models of care delivery. By being situated in the center of an academic hospital that primarily provides in-person care, the Garden Room makes video consulting easily accessible to healthcare providers. The involvement of end-users in the design process and consideration of human factors in the design enables the Garden Room to meet the needs of healthcare providers. The incorporation of natural elements, comfortable workspaces, and appropriate technologies in the physical space can create a positive environment for both healthcare providers and patients. However, the adoption and effectiveness of the Garden Room will also depend on the behavior and work processes of the healthcare professionals using it. Post-occupancy evaluation, behavioral and organizational measures are now needed to understand and further promote the adoption of video consulting among hospital caregivers. Finally, as the healthcare landscape continues to evolve, it is essential to continuously explore the potential for incorporating other telehealth solutions and to adapt the design of physical space accordingly to promote scalability and sustainability of the Garden Room.

## Data Availability

The raw data supporting the conclusions of this article will be made available by the authors, without undue reservation.
