# Supplementary material for: Designing a video consultation area for hybrid care delivery: the Garden Room with a view
Source: Front Digit Health. 2023 Jul 25;5:1198565. doi: 10.3389/fdgth.2023.1198565 (PMC10411545; doi:10.3389/fdgth.2023.1198565)

Supplementary Material

Designing a video consultation area for hybrid care delivery:
the garden room with a view

Merlijn Smits^1†^, PhD, Demi van Dalen^2†*^, MSc, Danny Popping^3^, René Bleker^2^, Martijn WJ Stommel^2^, MD, PhD, Harry van Goor^2^, MD, PhD

*** Correspondence:** Demi van Dalen: [demi.vandalen@radboudumc.nl](mailto:demi.vandalen@radboudumc.nl)

# Supplementary Figures and Tables

# Supplementary table 1. Identified end-users needs

| Consultation room set-up | Technology | Room availability |
| --- | --- | --- |
| Daylight(like) lighting | Adequate WiFi connection for using your own devices | Ability to make a reservation for a video consultation room |
| Lighting can be dimmed | Adequate mobile phone reception in order be accessible | Sufficient availability |
| Capacity for two persons in each cell | Frequent user gets own headset | Free walk-in/last minute facilitation |
| Height adjustable camera | Landline telephone in each box | General asset, not by specialty |
| Sufficient charging outlets | IT-support | Prevent peaks |
|  | PC with installed a video call system | Modularity |
|  | Virtual waiting area |  |

**Supplementary figure 2. Design video consultation box**
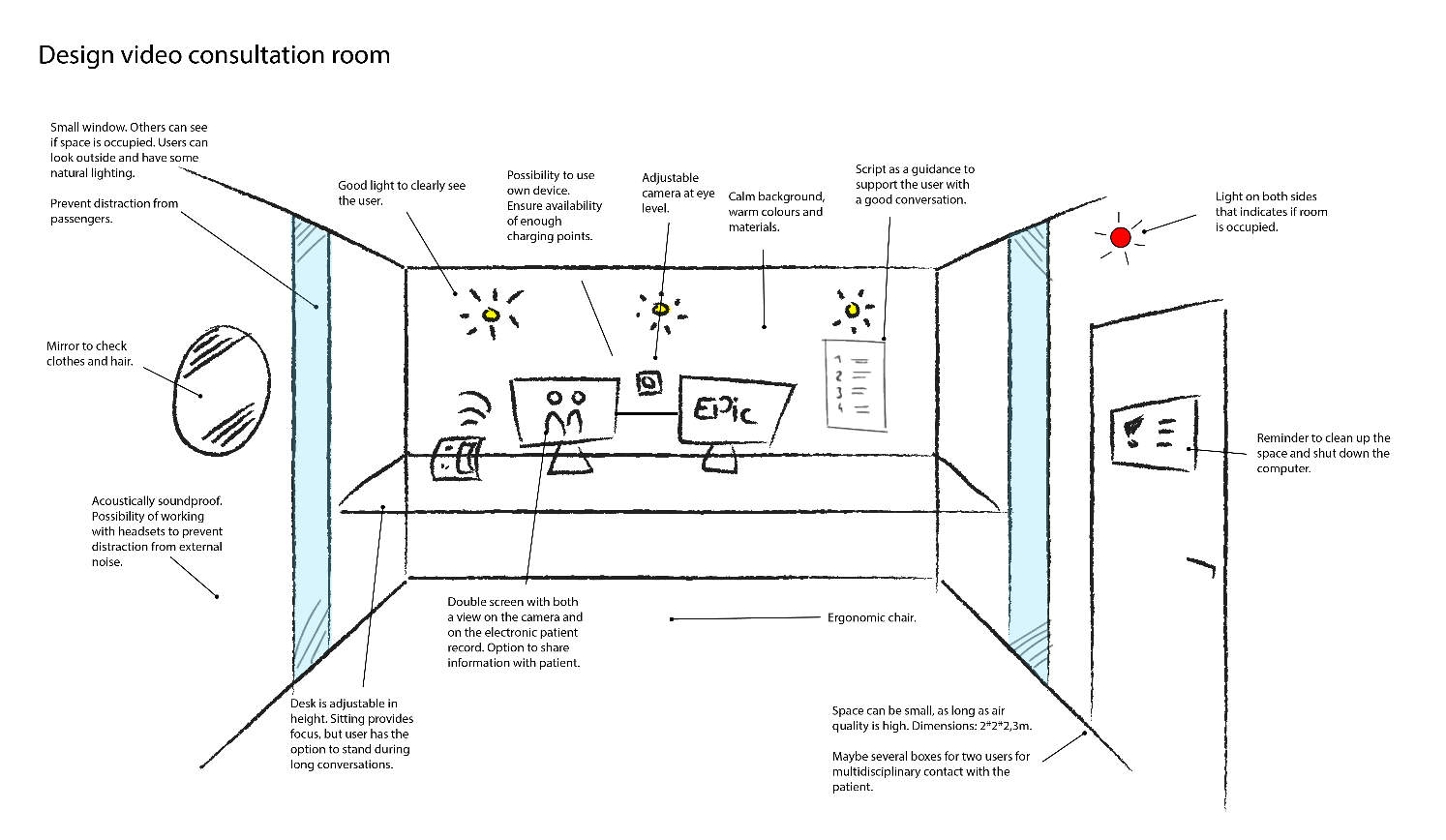

Supplement: Supplementary file 1 [file Datasheet1.docx]
